# The associations between body composition and vital capacity index of medical students in Shenyang of China: a cross-sectional survey

**DOI:** 10.1186/s12890-022-02176-8

**Published:** 2022-10-02

**Authors:** Han Zhang, Lu Sun, Ye Yu, Hong Xin, Li Wu, Fengmei Yang, Jie Liu, Zhuo Zhang

**Affiliations:** 1 School of Public Health, Shenyang Medical College, Shenyang, 110034 China; 2 Radiation Health Center, Liaoning Provincial Center for Disease Control and Prevention, Shenyang, 110015 China; 3 Physical Education Department, Shenyang Medical College, Shenyang, 110034 China

**Keywords:** Obesity, Fat mass, Body composition, Vital capacity index, Medical students

## Abstract

**Objectives:**

This study aimed to examine the associations between body composition and vital capacity index (VCI) among medical students of Shenyang, China.

**Study design:**

The design of this study is a cross-sectional study.

**Methods:**

Participants were 2063 individuals (17–25 years) from a medical college in Shenyang, who participated in this survey from April to May 2017. Height, weight, fat mass (FM), fat free mass (FFM), protein mass (PM), total body water (TBW), mineral mass (MM), vital capacity were measured, then BMI and VCI were calculated. Stepwise multiple linear regression analysis was used to evaluate the effect of body composition on VCI of participants in different genders. In addition, subgroup analysis was carried out according to BMI levels.

**Results:**

Male students showed significantly higher height, weight, BMI, FFM, PM, TBW, MM, VC, and VCI, but lower FM in comparison with female students. Stepwise multiple linear regression analysis showed that in both sexes FM was negatively correlated with VCI which represents pulmonary function (*r* < 0; *P* < 0.001). After dividing the whole participants by BMI, further correlation analysis showed FM was positively correlated with VCI only for male subgroups with BMI < 18.5 (*r* > 0; *P* = 0.050).

**Conclusion:**

Overall, FM is highly negatively correlated with the VCI of Chinese medical students of both genders. However, there was a positive correlation between FM and VCI among low-weight male students.

## Background

Obesity is becoming a global epidemic [1] and has been proved to be a risk factor for many diseases such as cancer, cardiovascular disease, diabetes and colitis [2–4]. According to the WHO criteria, Obesity was defined as BMI ≥ 30 kg/m^2^ [5, 6]. Generally speaking, as a result of increase in weight and body fat, obesity cause or aggravates the symptoms of some diseases. Respiratory movement is undertaken and carried out through the interaction of respiratory muscles, lungs and chest wall [7, 8], so fat thickening of chest wall increases respiratory resistance and then leads to the decline of lung function [9].

Interestingly, the results of current studies on the relationship between obesity and lung function are not consistent. On the one hand, obesity is a risk factor for chronic respiratory diseases such as chronic obstructive pulmonary disease (COPD), asthma and bronchitis [10–13]. On the other hand, obesity can reduce mortality in COPD, and provide a protective factor for the prognosis of many chronic respiratory diseases [14, 15]. How obesity can be a protective factor against chronic diseases? Some studies [16, 17] have shown that obesity may reduce inflammatory cytokines, including interleukin-6 and interleukin-8. In addition, obese patients have higher energy reserves than people of normal weight, so this may give obese patients a survival advantage in the event of ARDS [18]. Researchers have discussed the contradictory results of these two aspects and conclude that one of the possible reasons is that body mass index (BMI), the most widely used obesity index, cannot exactly show body composition status [14], thus easily giving rise to "obesity paradox" in those studies. The "obesity paradox " [14, 15] found in patients with chronic respiratory diseases means that in addition to BMI, other specific indicators related to body composition should be used for the relationship between body composition and lung function.

Body composition includes fat mass (FM), protein mass (PM), total body water (TBW) and mineral mass (MM) in the human body [19], and the measurement of body composition is closely related to evaluate the health situation of human. In recent years, more and more studies have shown that body composition indicators play an important role in the evaluation of pulmonary function [20–22]. With the development of body composition measurement technology, body composition has gradually become a focus point for study of lung function.

At present, most of the studies on body composition and cardiopulmonary function are limited to high-risk groups such as patients and the elderly [23, 24], while there are relatively few studies on healthy subjects, especially college students who are at a special stage. The college years is likely a critical period for risk of weight gain and body composition change among young-adults[25]. The abnormal changes of body composition and cardiopulmonary function in this stage are causing a range of adverse health effects on physical health of adults. Further studies into the body composition and lung function of this group are warranted.

Therefore, this study was conducted using the data of body composition and vital capacity index (VCI) collected from medical students in Shenyang with the objective to explore the relationship between body composition and VCI.

## Materials and methods

The cluster sampling survey method for a cross-sectional study was conducted in Shenyang, China from April to May 2017, and students were recruited from Shenyang Medical College, and the informed verbal consent of each participant was obtained. The Ethics Committee of Shenyang Medical college (grant number: 2017–006) has approved the informed verbal consent procedure for the study. The recruitment criteria were as follows: participants were functionally independent Chinese medical students over 17–25 years of age, who were in apparent good health. Participants were included if they did not meet any of the following exclusion criteria: (1) Participants with severe metabolic and respiratory diseases. (2) Participants who refused to participate in this study.

**Fig. 1:**
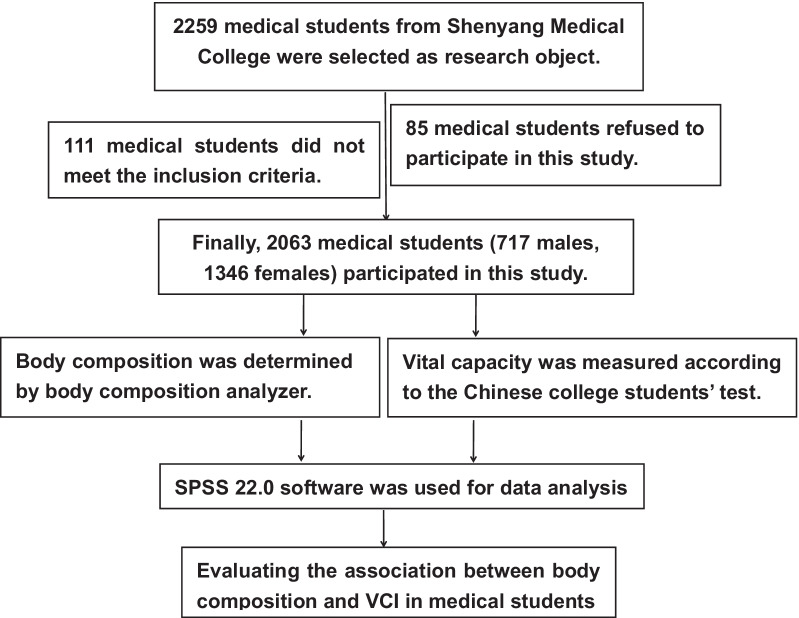
The flow chart of the study enrollment

After that, 2259 medical students recruited were measured for body composition, vital capacity (VC) and the corresponding data were collected. Of all these participants, 196 students without providing valid data were excluded during the test. In the end, 2063 participants in total were eventually eligible for the statistical analyses. The flow chart is shown in Fig. 1.

### Vital capacity test

VC was measured by using a spirometry meter (model number: WQS-8888, Wanqing Electronics, Shanghai, China) according to the guidance of the National Physical Health Test Standard [26]. In short, everyone stood with his feet flat on the floor, and took a deep breath, then exhaled steadily into the mouthpiece for as long as possible until there was no air left. The maximum value was recorded after three measurements. Since VC is affected by body shape such as height and weight, we employed the VCI with the consideration of the body mass, to represent the lung ventilation function: VCI = VC (ml) / weight (kg).

### Anthropometric measurement

Participants were asked to remove their shoes and coats before anthropometric measurement. The height was accurate to 0.1 cm, and the weight was accurate to 0.1 kg. BMI was calculated as body weight (in kilograms) divided by the square of height (in metres), as shown in the following formula: BMI = weight (kg) / height (m)^2^. Based on Chinese National Standard of BMI Classification [27], a BMI lower or equal to 18.5 kg/m^2^ is defined as underweight, between 18.5 kg/m^2^ and 23.9 kg/m^2^ is considered as normal weight for individuals. Overweight is defined as BMI between 24.0 and 27.9 kg/m^2^, and obesity is defined as BMI above 28.0 kg/m^2^.

### Body composition test

Body composition was determined by body composition analyzer (model number: BCA-1B Tsinghua Tongfang, Beijing, China) including FM, fat-free mass (FFM), PM, TBW and MM. After the preheating 30 min or above, all participants removed the coat and all metal products, stood barefoot parallel on the pedal electrode, grasped the hand-held electrode with both hands, looked forward, and remained motionless, then the arms were naturally separated from the body about 15°, raised the head. After that, the test results were transmitted to the computer for data processing.

### Statistical analysis

Statistical analysis was performed using SPSS version 22.0. Independent sample *t*-test was used to compare the two groups [9, 28]. To investigate associations between body composition (x) and VCI (y), stepwise linear regression analyses were conducted [29, 30]. Two regression models were created: a. unadjusted model**I**; b. model **II**adjusted for age and ethnicity of the medical students. The measurement data were expressed as mean ± standard deviation. All hypothesis tests were two-sided and a *p* < 0.05 was considered statistically significant.

## Results

### Participant characteristics

A total of 2063 participants were enrolled in this study. Of these, 717 male students were aged 17– 25 years and 1346 female students were aged 17– 24 years. Table 1 shows descriptive statistics for the participants grouped by sex. The age, height, weight, BMI, FFM, PM, TBW, MM, VC and VCI of male students were significantly higher than those of female students (*P* = 0.002 or *P* < 0.001), while FM was significantly lower than that of female students (*P* < 0.001).

**Table 1:** Characteristics of the participants

Characteristic	Total (2063)	Male (717)	Female (1346)	*P* value
Age (years)	20.398 ± 1.264	20.515 ± 1.248	20.337 ± 1.269	0.002
Height (cm)	167.182 ± 8.039	175.061 ± 6.027	162.985 ± 5.387	< 0.001
Weight (Kg)	61.412 ± 12.786	70.292 ± 13.406	56.682 ± 9.515	< 0.001
BMI (Kg/m^2^)	21.867 ± 3.597	22.896 ± 3.937	21.319 ± 3.273	< 0.001
FM (Kg)	14.831 ± 5.877	12.960 ± 6.099	15.828 ± 5.504	< 0.001
FFM (Kg)	46.584 ± 9.945	57.332 ± 8.115	40.859 ± 4.709	< 0.001
PM (Kg)	9.573 ± 2.071	11.810 ± 1.691	8.381 ± 0.982	< 0.001
TBW (Kg)	33.941 ± 7.339	41.873 ± 5.989	29.715 ± 3.474	< 0.001
MM (Kg)	3.070 ± 0.534	3.648 ± 0.436	2.763 ± 0.253	< 0.001
VC (ml)	3843.482 ± 1005.305	4883.842 ± 775.702	3289.293 ± 587.144	< 0.001
VCI (ml/kg)	63.811 ± 12.972	71.783 ± 12.494	59.564 ± 11.089	< 0.001

*BMI* Body mass index, *FM* Fat mass, *FFM* Fat-free mass, *PM* Protein mass, *TBW* Total body water, *MM* Mineral mass, *VC* Vital capacity, *VCI* Vital capacity index

### Correlations of body composition and VCI

**Fig. 2:**
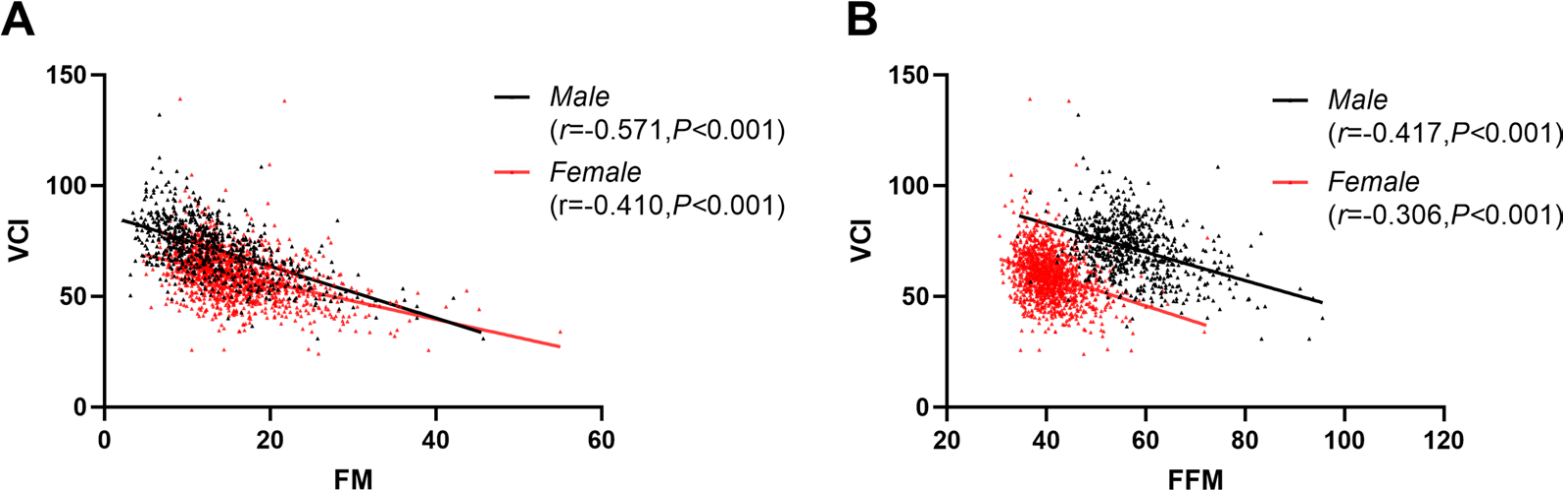
Scatter plot of the correlation between FM (**A**), FFM (**B**) and VCI (**A**–**B**)

In Fig. 2, Pearson’s correlation analysis showed, for male students, negative correlations between FM and VCI and between FFM and VCI, respectively (*r* < 0, *P* < 0.001). Female students showed similar result as male students, and FM and FFM were negatively correlated with VCI (*r* < 0, *P* < 0.001).

### Stepwise multiple regression between body composition and VCI

To further examine the relationship between body composition indices (FM, PM, TBW and MM) and VCI, two multiple regression models were developed. In Model I of Table 2, only FM was negatively associated with VCI (*P* < 0.001) in two sexes respectively, and other body composition indices had no statistically significant associations with VCI. In adjusted analysis of Model II, each unit (kg) increase in FM was associated with a 0.573 points (*P* < 0.001) decrease in VCI for male students, a 0.411 points (*P* < 0.001) decrease for female students.

**Table 2:** Stepwise multiple linear regression of VCI on body composition indices

			*B*(*95%CI*)	*β*	*S.E*	*t*	*P*	*Vif*
Male	Model I	FM	− 1.169(− 1.293,− 1.046)	− 0.571	0.063	− 18.591	< 0.001	1.000
	Model II	FM	− 1.173(− 1.296,− 1.050)	− 0.573	0.063	− 18.730	< 0.001	1.001
Female	Model I	FM	− 0.826(− 0.924,− 0.728)	− 0.410	0.050	− 16.479	< 0.001	1.000
	Model II	FM	− 0.827(− 0.926,− 0.729)	− 0.411	0.050	− 16.519	< 0.001	1.000

*FM* Fat mass. Model II was adjusted for student’s age and nationality

### Correlation analysis between FM and VCI in the different BMI groups

**Fig.3:**
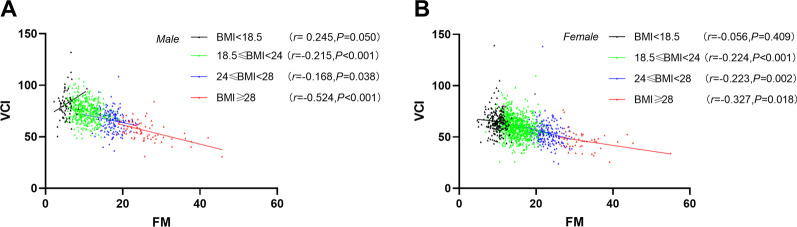
Scatter plot of the Pearson correlation between FM and VCI in different BMI groups

In Fig. 3, further Pearson correlation analysis showed that there was a significant negative correlation between FM and VCI in different BMI groups of male and female students with BMI > 18.5 kg/m^2^, respectively. However, FM was positively correlated with VCI only in male students with BMI < 18.5 kg/m^2^ (*P* = 0.050). In particular, body composition analysis showed that among the students with BMI less than 18.5, the FM (9.78 ± 1.58 kg) of female students was significantly higher than that (5.36 ± 1.38 kg) of male students (*P* < 0.001).

## Discussion

In this study, a cross-sectional study was conducted to evaluate the relationships between body composition and VCI of medical students in China. By comparing the body composition, the results showed that FFM and VCI of male students were higher than those of females, while FM was lower than those of female students. This seems to be related to the physiological condition of both sexes, due to the role of androgen, the body FFM of men increases significantly, while for women estrogen plays an important role in the increase of FM [31].

After Pearson correlation and further stepwise regression analysis, we found that among the subjects of different genders, only FM was negatively correlated with VCI, and the negative correlation still existed after adjusting for confounding factors. Other studies, when taken together, also support this result of the correlation [32–34]. Although the mechanism underlying the association has not been elucidated, considering comprehensively, and increased FM can potentially cause decreased VCI in below ways: first of all, the increase of FM leads to weight gain, and the body overcomes its own load, which will cause an increase in vital capacity after a period of time [35]. Secondly, the adipose tissue of the chest and abdomen may lead to a decrease in the compliance of chest and lung tissue, at the same time, the structure of respiratory muscle and trachea is abnormal, and the ventilatory capacity of lung tissue is limited. Previous studies have shown decreased chest wall compliance in obese patients who are awake and breathing spontaneously, which supports our speculation [36–38]. The third possible explanation is that adipose tissue can be involved in the secretion of a variety of inflammatory factors, since it is considered one of the largest endocrine organs in the body [39, 40]. In the study of body composition, adipose tissue is the site of the early link between inflammation and obesity. Hotamisligil et al. [41] found that TNF- *α* was over-expressed in adipose tissue of obese mice, providing the first link between obesity, diabetes and chronic inflammation. Later studies confirmed that there are over-expressed pro-inflammatory and inflammatory mediators in adipose tissue of obese patients, such as TNF-*α*, IL-6, monocyte chemoattractant protein-1, and macrophages infiltrate adipose tissue [42]. It is worth noting that a variety of cytokines produced by fat-induced inflammation are also detected many times in lung diseases, so adipose tissue may regulate the endocrine system, lead to chronic inflammation, and then cause or aggravate the occurrence and development of abnormal lung function [43–45].

After determining that FM is the main influencing factor of VCI, we further divided our subject population into four groups to explore the relationship between FM and VCI of participants from different BMI levels. Of note, we also showed that there was a positive correlation between FM and VCI in male students with BMI < 18.5. Contrary to our results, a survey showed that fat tissue was not correlated with lung function in males [46]. But the study did not survey participants whose BMI was less than 18.5. This may be one of the reasons for the different results.

This result may provide a new point of view that FM has a protective effect on pulmonary ventilatory function in low-weight male students. An interesting physiological explanation may be that a protective increase in lung recoil has been found in patients with higher BMI [47], while the loss of lung recoil is often associated with decreased lung function and COPD progression [48]. Since the FM varies in men and women, it is reasonable that gender difference in FM contributes differently to lung functions for two sexes with low weight, in addition to the gender difference in hormones.

Adipose tissue makes up around 15–20% of the body weight of an average person, which actively participates in the functioning of the body. However, unusually high levels of this type of tissue have been clearly linked with health problems including non-communicable disorders as well as cardiovascular disorders [49, 50].

In recent years, the associations of obesity with reduced pulmonary function and chronic airway disease have received considerable attention [51–53]. In particular, a close relationship between obesity and VCI has been suggested [32]. However, evidence is limited with regard to whether body composition is associated with VCI among the Chinese medical students. This study went some way to filling a gap in such research.

This study has several limitations. First, this study is a cross-sectional study which can only explore the correlation between body composition and lung function rather than causality. Also, this study clarified the relationship between FM and VCI, but neglected the effects of adipose tissue in different parts of the body on lung function; thus, we cannot confidently extrapolate findings to how body fat distribution affects VCI in Chinese medical students. Finally, the subjects of this study are only Han and Manchu medical students, which may lead to the results of the study can not be extrapolated to other ethnic groups. Here, we also suggest to strengthen the research on the correlation between body composition and pulmonary ventilation function of thin and weak groups, in order to better provide effective guidance for such groups.

## Conclusion

Generally speaking, there was a negative correlation between FM and VCI in Chinese medical students with both sexes. However, there was a positive correlation between FM and VCI among low-weight male students.

## Data Availability

The datasets generated and/or analyzed during the current study are not publicly available due to limitations of ethical approval involving the student data and anonymity but are available from the corresponding author on reasonable request.

## Abbreviations

BMI: Body mass index
COPD: Chronic obstructive pulmonary disease
FFM: Fat free mass
FM: Fat mass
MM: Mineral mass
PM: Protein mass
TBW: Total body water
VC: Vital capacity
VCI: Vital capacity index

## References

[1] Laving A, Hussain S, Atieno D (2018). Overnutrition: does complementary feeding play a role?. Annal Nutr Metab.

[2] Fu W, Cao S, Liu B, Li H, Song F, Gan Y, Li W, Opoku S, Yan S, Yue W (2018). Association of general and central adiposity with blood pressure among Chinese adults: results from the China national stroke prevention project. J Hypertens.

[3] Yaghootkar H, Lotta LA, Tyrrell J, Smit RA, Jones SE, Donnelly L, Beaumont R, Campbell A, Tuke MA, Hayward C (2016). Genetic evidence for a link between favorable adiposity and lower risk of type 2 diabetes, hypertension, and heart disease. Diabetes.

[4] Kolb R, Sutterwala FS, Zhang W (2016). Obesity and cancer: inflammation bridges the two. Curr Opin Pharmacol.

[5] WHO Expert Consultation. Appropriate body-mass index for Asian populations and its implications for policy and intervention strategies. Lancet (London, England) 2004;363(9403):157–163. 10.1016/S0140-6736(03)15268-3 14726171

[6] WHO Consultation on Obesity (1999: Geneva, Switzerland) & World Health Organization. Obesity: preventing and managing the global epidemic. Report of a WHO consultation. World health organization technical report series 2000; 894(i–xii), 1–253. 11234459

[7] Segizbaeva MO, Timofeev NN, Donina ZhA, Kur'yanovich EN, Aleksandrova NP (2015). Effects of inspiratory muscle training on resistance to fatigue of respiratory muscles during exhaustive exercise. Adv Exp Med Biol.

[8] Nava S, Ambrosino N, Crotti P, Fracchia C, Rampulla C (1993). Recruitment of some respiratory muscles during three maximal inspiratory manoeuvres. Thorax.

[9] Al Ghobain M (2012). The effect of obesity on spirometry tests among healthy non-smoking adults. BMC Pulm Med.

[10] Hanson C, Rutten EP, Wouters EF, Rennard S (2014). Influence of diet and obesity on COPD development and outcomes. Int J Chron Obstruct Pulmon Dis.

[11] Peters U, Dixon AE, Forno E (2018). Obesity and asthma. J Allergy Clin Immunol.

[12] Lee YL, Chen Y-C, Chen Y-A (2012). Obesity and the occurrence of bronchitis in adolescents. Obesity.

[13] Zewari S, Vos P, van den Elshout F, Dekhuijzen R, Heijdra Y (2017). Obesity in COPD: revealed and unrevealed issues. COPD.

[14] Spelta F, Fratta Pasini AM, Cazzoletti L, Ferrari M (2018). Body weight and mortality in COPD: focus on the obesity paradox. Eat Weight Disord.

[15] Yano C, Kawayama T, Kinoshita T, Tokunaga Y, Sasaki J, Sakazaki Y, Matsuoka M, Imaoka H, Nishiyama M, Matsunaga K (2021). Overweight improves long-term survival in Japanese patients with asthma. Allergol Int.

[16] Stapleton R, Dixon A, Parsons P, Ware L, Suratt B (2010). The association between BMI and plasma cytokine levels in patients with acute lung injury. Chest.

[17] Maia L, Cruz F, de Oliveira M, Samary C, Fernandes M, Trivelin S, Rocha N, Gama de Abreu M, Pelosi P, Silva P et al: Effects of obesity on pulmonary inflammation and remodeling in experimental moderate acute lung injury. Front Immunol 2019; 10.3389/fimmu.2019.01215. 10.3389/fimmu.2019.01215 PMC6593291 31275296

[18] Ng P, Eikermann M (2017). The obesity conundrum in sepsis. BMC Anesthesiol.

[19] Kuriyan R (2018). Body composition techniques. Indian J Med Res.

[20] Gonzalez-Barcala FJ, Takkouche B, Valdes L, Leis R, Alvarez-Calderon P, Cabanas R, Rodriguez Suarez JR, Tojo R (2007). Body composition and respiratory function in healthy non-obese children. Pediatr Int.

[21] Peralta G, Fuertes E, Granell R, Mahmoud O, Roda C, Serra I, Jarvis D, Henderson J, Garcia-Aymerich J (2019). Childhood body composition trajectories and adolescent lung function findings from the ALSPAC study. Am J Respir Crit Care Med.

[22] Skrypnik D, Bogdanski P, Madry E, Karolkiewicz J, Ratajczak M, Krysciak J, Pupek-Musialik D, Walkowiak J (2015). Effects of endurance and endurance strength training on body composition and physical capacity in women with abdominal obesity. Obes Facts.

[23] Karstoft K, Winding K, Knudsen SH, Nielsen JS, Thomsen C, Pedersen BK, Solomon TP (2013). The effects of free-living interval-walking training on glycemic control, body composition, and physical fitness in type 2 diabetic patients: a randomized, controlled trial. Diabetes Care.

[24] Park W, Jung WS, Hong K, Kim YY, Kim SW, Park HY (2020). Effects of moderate combined resistance- and aerobic-exercise for 12 weeks on body composition cardiometabolic risk factors blood pressure arterial stiffness and physical functions among obese older men: a pilot study. Int J Environ Res Public Health.

[25] Deliens T, Clarys P, De Bourdeaudhuij I, Deforche B (2014). Determinants of eating behaviour in university students: a qualitative study using focus group discussions. BMC Public Health.

[26] Zhang F, Bi C, Yin X, Chen Q, Li Y, Liu Y, Zhang T, Li M, Sun Y, Yang X (2021). Physical fitness reference standards for Chinese children and adolescents. Sci Rep.

[27] Zhu Q, Huang B, Li Q, Huang L, Shu W, Xu L, Deng Q, Ye Z, Li C, Liu P (2020). Body mass index and waist-to-hip ratio misclassification of overweight and obesity in Chinese military personnel. J Physiol Anthropol.

[28] Ortiz-Prado E, Encalada S, Mosquera J, Simbaña-Rivera K, Gomez-Barreno L, Duta D, Ochoa I, Izquierdo-Condoy J, Vasconez E, Burgos G (2022). A comparative analysis of lung function and spirometry parameters in genotype-controlled natives living at low and high altitude. BMC Pulm Med.

[29] Garvey M, Shi L, Gona P, Troped P, Camhi S (2021). Age sex and race/ethnicity associations between fat mass and lean mass with bone mineral density: NHANES data. Int J Environ Res Publ Health.

[30] Xiao Z, Xu H (2020). Gender-specific body composition relationships between adipose tissue distribution and peak bone mineral density in young Chinese adults. Biomed Res Int.

[31] Bredella M (2017). Sex differences in body composition. Adv Exp Med Biol.

[32] Huang L, Ye Z, Lu J, Kong C, Zhu Q, Huang B, Wang Z, Xu L, Deng Q, Gong J (2019). Effects of fat distribution on lung function in young adults. J Physiol Anthropol.

[33] Maiolo C, Mohamed E, Carbonelli M. Body composition and respiratory function. Acta diabetologica 2003;40:s32–s38. 10.1007/s00592-003-0023-0 14618430

[34] Kawabata R, Soma Y, Kudo Y, Yokoyama J, Shimizu H, Akaike A, Suzuki D, Katsuragi Y, Totsuka M, Nakaji S (2020). Relationships between body composition and pulmonary function in a community-dwelling population in Japan. PLoS ONE.

[35] Ma C, Wang Y, Xue M (2019). Correlations of severity of asthma in children with body mass index, adiponectin and leptin. J Clin Lab Anal.

[36] Canoy D, Luben R, Welch A, Bingham S, Wareham N, Day N, Khaw K (2004). Abdominal obesity and respiratory function in men and women in the EPIC-Norfolk Study. UK Am J Epidemiol.

[37] Naimark A (1960). Cherniack R compliance of the respiratory system and its components in health and obesity. J Appl Physiol.

[38] Chen Y, Rennie D, Cormier Y, Dosman J (2007). Waist circumference is associated with pulmonary function in normal-weight, overweight, and obese subjects. Am J Clin Nutr.

[39] Kershaw E, Flier J (2004). Adipose tissue as an endocrine organ. J Clin Endocrinol Metab.

[40] Trayhurn P, Beattie J (2001). Physiological role of adipose tissue: white adipose tissue as an endocrine and secretory organ. Proc Nutr Soc.

[41] Hotamisligil G (2006). Inflammation and metabolic disorders. Nature.

[42] Nishimura S, Manabe I, Nagasaki M, Eto K, Yamashita H, Ohsugi M, Otsu M, Hara K, Ueki K, Sugiura S (2009). CD8+ effector T cells contribute to macrophage recruitment and adipose tissue inflammation in obesity. Nat Med.

[43] Brown SD, Brown LA, Stephenson S, Dodds JC, Douglas SL, Qu H, Fitzpatrick AM (2015). Characterization of a high TNF-alpha phenotype in children with moderate-to-severe asthma. J Allergy Clin Immunol.

[44] Singh S, Verma S, Kumar S, Ahmad M, Nischal A, Singh S, Dixit R (2018). Correlation of severity of chronic obstructive pulmonary disease with potential biomarkers. Immunol Lett.

[45] Gou X, Zhang Q, More S, Bamunuarachchi G, Liang Y, Haider Khan F, Maranville R, Zuniga E, Wang C, Liu L (2019). Repeated exposure to streptococcus pneumoniae exacerbates chronic obstructive pulmonary disease. Am J Pathol.

[46] Scott H, Gibson P, Garg M, Pretto J, Morgan P, Callister R, Wood L (2012). Relationship between body composition, inflammation and lung function in overweight and obese asthma. Respir Res.

[47] Pellegrino R, Gobbi A, Antonelli A, Torchio R, Gulotta C, Pellegrino GM, Dellaca R, Hyatt RE, Brusasco V (2014). Ventilation heterogeneity in obesity. J Appl Physiol.

[48] O'Donnell DE, Laveneziana P (2006). The clinical importance of dynamic lung hyperinflation in COPD. COPD.

[49] Dhawan D, Sharma S (2020). Abdominal obesity, adipokines and non-communicable diseases. J Steroid Biochem Mol Biol.

[50] Oikonomou E, Antoniades C (2019). The role of adipose tissue in cardiovascular health and disease. Nat Rev Cardiol.

[51] Zhu J, Zhao Z, Wu B, Shi Z, Nie Q, Fu Z, Zeng Z, Hu W, Dong M, Xiong M (2020). Effect of body mass index on lung function in chinese patients with chronic obstructive pulmonary disease: a multicenter cross-sectional study. Int J Chron Obstruct Pulmon Dis.

[52] Dixon A, Peters U (2018). The effect of obesity on lung function. Expert Rev Respir Med.

[53] Littleton S, Tulaimat A (2017). The effects of obesity on lung volumes and oxygenation. Respir Med.

